# A conversation with Stanley Prusiner

**DOI:** 10.1172/JCI183743

**Published:** 2024-07-15

**Authors:** Ushma S. Neill

The discovery that a protein alone could be infectious, proposed by Stanley Prusiner of the University of California San Francisco (UCSF), was considered heretical in 1982. Now considered orthodoxy, at that time, scientists thought that the only infectious agents were bacteria, viruses, fungi, and parasites. We now know that these proteins, termed prions, which acquire an alternative shape and coax their neighboring proteins to do the same, undergird a variety of neurodegenerative diseases. For his dogma-shattering work, Dr. Prusiner (Figure 1) has been widely recognized, including with the 1997 Nobel Prize in Physiology or Medicine. Watch the whole interview at https://www.jci.org/videos/cgms for his stories about when he almost left science to sell real estate and how he thought the call awarding him the Nobel Prize might have been a prank.

*JCI*: What were you like as a child?

Prusiner: I grew up first in Des Moines, Iowa, during World War II, then for a while in Cincinnati, Ohio. We moved back and forth. My father was an architect, and he never made a very good living. He was always upset that things weren’t better, and as very frequently happened in the world of Jews, my mother and father pushed my brother and me to become doctors. I didn’t get the bug and the excitement of science until I matriculated to the University of Pennsylvania.

*JCI*: How did you choose chemistry as your major?

Prusiner: When I was in high school, my homeroom teacher was a chemist named Jake Skilken. He taught a class I wanted to take — the advanced chemistry course. To get in, you had to pass a physics test, and several of us answered some questions incorrectly in his estimation, but the physics problem he asked was not well described. So I wasn’t allowed to take his course and ended up tutoring numerous students in the regular chemistry track.

*JCI*: So you majored in chemistry in college out of spite?

Prusiner: Nah, chemistry was always easy for me, and I knew that I wanted to go to medical school as was ordained by my parents. And I knew that I needed to do some research along the way. My aunt, who lived in Philadelphia and knew many influential people, helped me by speaking to the VP for Medical Affairs at Penn, Isidore Ravdin. After Ravdin operated on President Eisenhower’s bowel obstruction due to regional enteritis, he became very well known. Ironically, I never met Ravdin, but I was extremely fortunate that Ravdin connected me with a young assistant professor of surgery named Sidney Wolfson. To my great fortune, I undertook a research project with Wolfson, who showed me how to read and write a scientific paper and how to think about science. I owe my career to him because he made science so exciting for me. While working with Wolfson, I began to puzzle about what happens during hibernation in hamsters. I began focusing on brown adipose tissue and wondered if the increased oxidation rate in brown fat might be responsible for warming up the hamsters and asked Wolfson if I might be able to work with the legendary Penn biochemist Britton Chance. I explained to Chance that I wanted to use the instrument that he had designed for measuring fluorescence on the surface of organs. After a few minutes, Chance asked me when I wanted to start my studies with him. Soon, I found a cold room that was available for nine months while a faculty member was on sabbatical. Next, I bought two dozen young Syrian hamsters and let them live in the cold room for 8 to 12 weeks before they hibernated. I used Chance’s instrument to measure the surface fluorescence; that increased dramatically, confirming my hypothesis about the oxidation rate. It wasn’t anything earth shattering, but it was great for me because I learned a lot.

Later in medical school (still at UPenn), my mother had a discussion with her friend, who worked at the NIH. At that time, the Vietnam War was beginning to expand using thousands of conscripted young American men. Her friend suggested that I should apply to the NIH to expand my interest in research and ideally not go to Vietnam. So that’s the next chapter.

*JCI*: You decided to do one intern year at UCSF before the research years at NIH?

Prusiner: I didn’t decide anything. The NIH decided that I had to do the internship before coming. I would have punted any further study of clinical medicine and focused on my science. But that was not to be. I chose UCSF in large part because I spent much of my fourth year in medical school in Stockholm doing research on brown adipose tissue. I had a preliminary interview at Harvard, but they were not interested in me if I wasn’t able show up for their mega interview in Boston. I didn’t have enough money to fly from Stockholm to Boston, so that was the end of that. So that’s how I ended up in San Francisco, as UCSF didn’t need me to fly there for an interview.

The internship was not easy; I was on call every other night, but I got through it and then landed at the NIH where I worked with Earl Stadtman, an extraordinary biochemist. He taught me how to do scientific investigation. He set a wonderful example for me to follow. A decade earlier, he discovered the modification of an enzyme called glutamine synthetase through careful analysis and by proving his hypothesis using half a dozen different experimental approaches. As I studied his papers, I found myself muttering, “One way would have been enough.” But that’s not what he was doing; I realized he didn’t want to be wrong. He wanted to build a solid foundation so he could move from one step to another and onto the next step. This was an unbelievably important lesson for me personally as my life unfolded into the scrapie/prion story.

*JCI*: When you returned to UCSF for your neurology residency, within the first couple of months, you encountered a pivotal patient that impacted the rest of your career. A woman with Creutzfeldt-Jakob disease (CJD).

Prusiner: I didn’t know what was going on with this patient, but the attending, a British-trained neurologist, said the woman probably had CJD. When I looked it up, it made a lot of sense. We had a few discussions about the patient, who was getting progressively worse, and I watched her deteriorate day after day. In the second month of her stay, an attending physician named Dick Baringer, who was a herpes virologist and neurologist, knew the whole story of kuru and could talk about it at length. Over the course of the six months before my patient eventually died, I witnessed the unremitting progression of her illness. I started to read about the field: there was a lot of literature, but it wasn’t the kind of literature I coveted because it was not about the chemistry; it was about the neurology and the transmissibility of diseases like kuru and CJD. This was exceedingly interesting. There were a lot of hurdles, but the science was so fascinating. What was this thing? I remember thinking maybe I could come up with some better approach to treating patients like her.

*JCI*: You established your lab at UCSF and had some early funding and publication success, though I note that it was largely centered on the choroid plexus.

Prusiner: The stuff on the choroid plexus was because I needed a project that could get funded by the NIH. There is an enzyme called γ glutamyl transpeptidase, and I thought that the choroid plexus might be an area where it is produced. It was hard to study the choroid plexus because it was so small in rodents, but there was a slaughterhouse just across the Bay where they would give me the choroid plexus from the brains of slaughtered cows. Ironically, I didn’t even want the cow brains at that time. These investigations were the basis for my first NIH grant as well as an NIH career development award. Shortly afterwards, I was anointed with a Howard Hughes Medical Investigator (HHMI) award, but after five years, some of the Hughes administrators decided that my ideas about scrapie were very unlikely to prove viable. This was enough for the UCSF chair of neurology to become convinced I was truly crazy, just as everyone else had thought for a long time. Most people remained convinced that scrapie and CJD were caused by “slow viruses” and that my ideas about an infectious protein were crazy. Yet no one had identified either the putative scrapie or CJD viruses. Though rejected by HHMI as well as by virologists, neurologists, and many other medical scientists, I refused to have my enthusiasm diminished for solving the scrapie prion problem.

*JCI*: As you were ready to publish the work showing that scrapie was not caused by a virus and that it absolutely was a protein, there was a handling editor that sat on it?

Prusiner: Eleanor Butts, the handling editor at *Science*, had a reputation for doing just this kind of thing. She was worried that my paper might not be correct and that *Science* would have a black eye. But at the same time, the UCSF chancellor at that time (Frank Sooy) was approached by David Perlman, the science writer for the *San Francisco Chronicle* newspaper. I had bumped into Perlman at the San Francisco airport, and he was interested in my work, so he asked Sooy if he could do an article focused on my work. It was the tenth anniversary of UCSF (UCSF had been part of Berkeley for a long time), and Sooy wanted a big splash on how great the research was on our campus. Once Perlman had an article that he was pretty far along with, he called me to say it would appear the next day on the front page of the *Chronicle*. *Science* had to publish it then. Next, the world went crazy because suddenly there was a new word: prion. A lot of reporters got excited about it and wanted to know more about prions.

*JCI*: The story of you coining the term “prion” is also really fascinating. But beyond that, the reaction to the new term and to your findings was particularly savage.

Prusiner: The coining of the term “prion” goes back to my aunt and her friend since she was three. My aunt’s friend married a man named Frank Westheimer, who was a famous Harvard chemist. When Westheimer was a visiting professor at UCSF, I set up a time to meet with him, and after telling him the whole story, he said, “Stan, this is really important; you’ve got to come up with a great word for this infectious protein. Don’t mess this up, because if you don’t get this right, someone else will, and then they will get the lion’s share of the credit.”

I thought I could find a Latin or Greek professor at Berkeley who could help come up with a word, but that didn’t happen, so I sat there with all these words, like “protein” and “infectious” and “agent,” and would just pick letters and try to put some minor logic together.

One day I was eating at the now defunct UCSF Faculty Club, and I wrote down P-R-I-O-N, and I said, “Oh, that looks nice.” My key was to have two syllables, have it short, and to have protein at the beginning. I left most of my tuna sandwich sitting there, but it wasn’t very good anyway. I walked across the street, and I went up to the seventh floor where my labs were. I had this huge dictionary and looked up “prion,” and it listed a bird in the South Pacific that travels between Tasmania and Antarctica. I figured nobody had ever heard of this bird, so I moved forward with prion.

Because I put a label on something that was totally new, I pissed off a lot of people. Beyond that, there were lots of people who had been studying in the scrapie field for many years; they were angry because all of a sudden, I grabbed all the credit and they were left saying, well, I don’t know what they said, but it wasn’t very nice. They also said I should have had a convocation of a large number of people who could have all gotten together and come up with some word like “agent.” When people kept attacking me, I just kept my head down and kept putting out the data to prove the prion hypothesis; some weren’t just angry about the new term — they didn’t believe it wasn’t a virus.

*JCI*: Did winning the Nobel silence your skeptics?

Prusiner: No, it made them so much angrier. “How the hell did he get this prize? And he got it alone. How come I wasn’t part of that?” They were just really, really angry.

The prize did help just by being there. It was a stamp of approval that made the path to funding clear. People weren’t worried anymore that I was some charlatan. It didn’t help me get on a bus for free or on an airplane for nothing. When I was at the NIH, I could travel anywhere in the country for a dollar after I put on my Public Health Service uniform; however, the prize did have other perks.

*JCI*: If you could not be a physician or a scientist, do you think there’s another vocation that could have kept you as happy?

Prusiner: No, not even being president of the United States. What a privilege I’ve had. There’s nothing that even approaches the ability to do science, to have a lot of wonderful colleagues, to have something to talk about that’s meaningful when you talk to other people. Something to think about when you go to sleep, something that you think about waking up, and to have a problem which takes a while to solve; that’s really wonderful.

No, this is a true fairy tale.

## Figures and Tables

**Figure 1:**
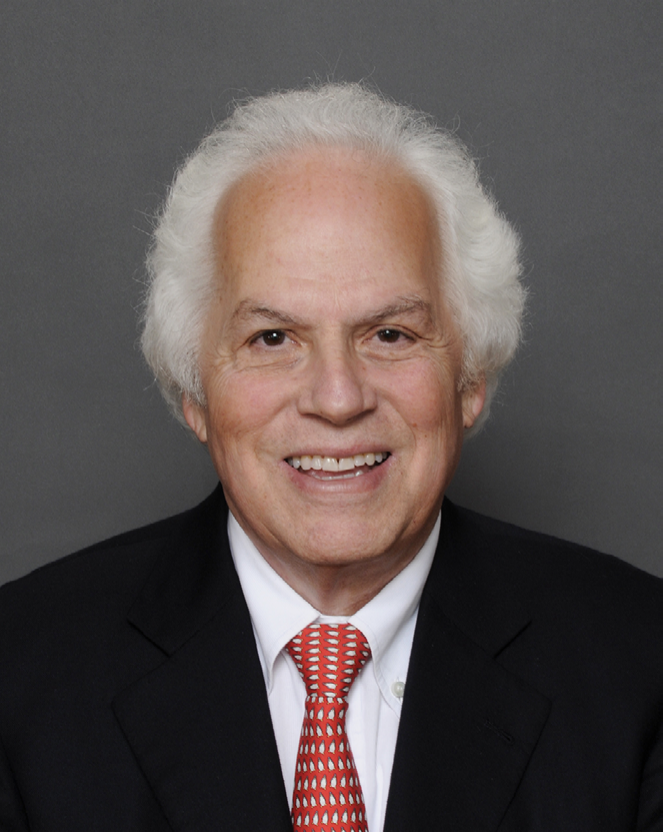
Stanley Prusiner. Image credit: Russ Fischella.

## Version 1 07/15/2024

Electronic publication

